# Complete genome sequence of *Marinomonas posidonica* type strain (IVIA-Po-181^T^)

**DOI:** 10.4056/sigs.2976373

**Published:** 2012-09-27

**Authors:** Patricia Lucas-Elío, Lynne Goodwin, Tanja Woyke, Sam Pitluck, Matt Nolan, Nikos C. Kyrpides, Janine C. Detter, Alex Copeland, Megan Lu, David Bruce, Chris Detter, Roxanne Tapia, Shunsheng Han, Miriam L. Land, Natalia Ivanova, Natalia Mikhailova, Andrew W. B. Johnston, Antonio Sanchez-Amat

**Affiliations:** 1 Department of Genetics and Microbiology, Regional Campus of International Excellence “Campus Mare Nostrum”,University of Murcia, Murcia, Spain; 2 DOE Joint Genome Institute, Walnut Creek, California, USA.; 3 Los Alamos National Laboratory, Bioscience Division, Los Alamos, New Mexico, USA; 4 Oak Ridge National Laboratory, Oak Ridge, Tennessee, USA; 5 School of Biological Sciences, University of East Anglia, Norwich Research Park, Norwich,UK

**Keywords:** Aerobic, Gram-negative, marine, plant-associated

## Abstract

*Marinomonas posidonica* IVIA-Po-181^T^ Lucas-Elío *et al.* 2011 belongs to the family *Oceanospirillaceae* within the phylum *Proteobacteria*. Different species of the genus *Marinomonas* can be readily isolated from the seagrass *Posidonia oceanica*. *M. posidonica* is among the most abundant species of the genus detected in the cultured microbiota of *P. oceanica,* suggesting a close relationship with this plant, which has a great ecological value in the Mediterranean Sea, covering an estimated surface of 38,000 Km^2^. Here we describe the genomic features of *M. posidonica*. The 3,899,940 bp long genome harbors 3,544 protein-coding genes and 107 RNA genes and is a part of the ***G****enomic* ***E****ncyclopedia of* ***Bacteria**** and* ***Archaea***** project.

## Introduction

Strain IVIA-Po-181^T^ is the type strain of *Marinomonas posidonica,* which belongs to the order *Oceanospirillales* within the class *Gammaproteobacteria*. Microorganisms belonging to the genus *Marinomonas* can be readily isolated from different parts of the marine angiosperm *Posidonia oceanica,* and many different *Marinomonas* species have been isolated from it [1,2]. The data obtained so far indicate that among the culturable microbiota in the phylloplane and rhizoplane of this plant, the closely related *M. posidonica* and *M. aquiplantarum* are the most abundant species [2]. *P. oceanica* (known commonly as Mediterranean tapeweed or Neptune Grass), is of great ecological value in the Mediterranean Sea. Recently, it has been estimated that large clones of this slow-growing plant can spread over several kilometers and can be several thousand years old [3]. The long lifespan of this plant and the abundance of *M. posidonica* in its microbiota suggest the possibility of an ancient relationship between them. In fact, *M. posidonica* has been shown to have a positive effect on the growth of *P. oceanica* seeds [4]. Here we describe the complete genomic sequencing and annotation of *M. posidonica* IVIA-Po-181^T^, along with some relevant features of this microorganism.

## Classification and features

**Figure 1:**
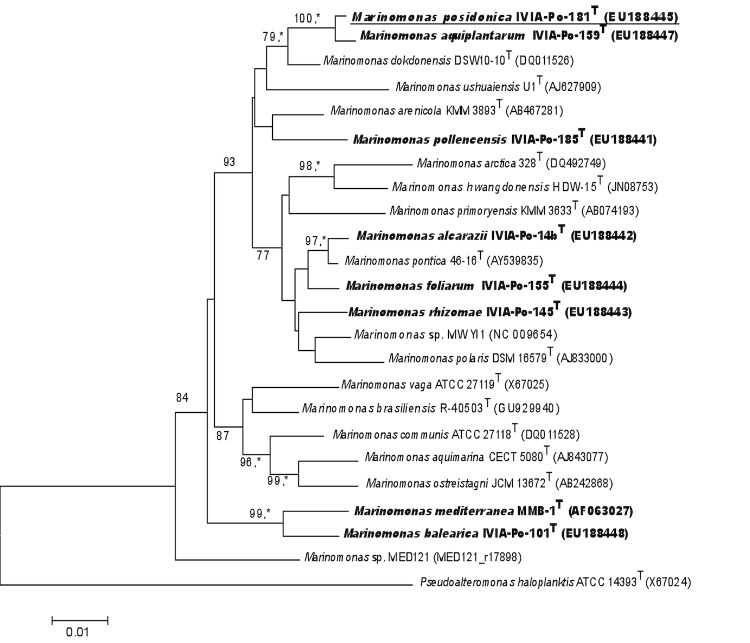
Phylogenetic tree highlighting the position of *Marinomonas posidonica* IVIA-Po-181^T^ in relation to other type and non-type strains within the genus *Marinomonas*. The species that have been isolated from *Posidonia oceanica* are in bold. The tree was generated using the program MEGA version 4 [5]. The sequences were aligned using the CLUSTAL W program within MEGA software. The tree was generated using the neighbor-joining method. Asterisks indicate branches that were also identified by the maximum-parsimony method. Numbers at branches indicate bootstrap values from 1,000 replicates. *P. haloplanktis* (X67024) was used as an outgroup.

*M. posidonica* IVIA-Po-181^T^ contains 8 copies of the 16S rRNA gene. The only observed difference among these is that the nucleotide at position 454 can be either T (in 6 of the copies) or C (two copies). The phylogenetic neighborhood of *M. posidonica* IVIA-Po-181^T^ in a tree based on 16S rRNA sequences is shown in Figure 1.

**Figure 2:**
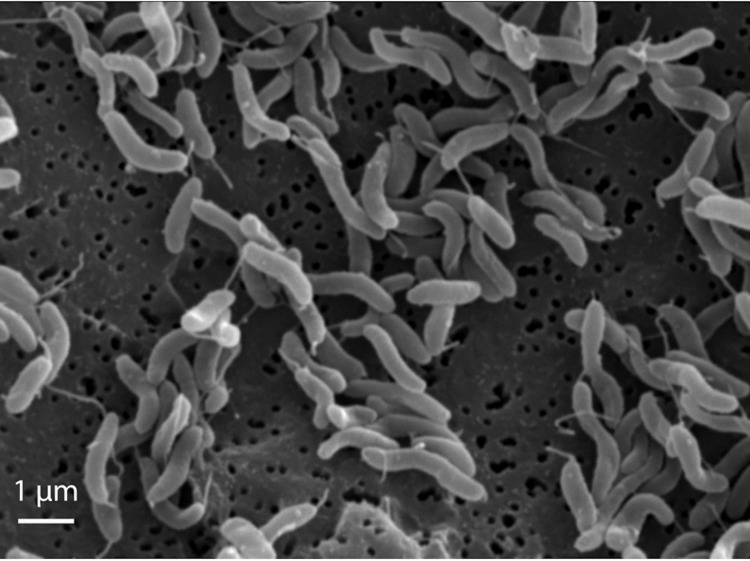
Electron micrograph of *M. posidonica* IVIA-Po-181^T^.

The cells of *M. posidonica* IVIA-Po-181^T^ are helical with rounded ends. Cell lengths and widths are approximately 1.3-2.1 and 0.4 μm respectively (Figure 2, Table 1). Strain IVIA-Po-181^T^ is motile by a single polar flagellum [2] (Figure 2). Colonies in complex medium, such as marine 2216 agar, do not show pigmentation. A Na^+^ concentration greater than 0.5% is required for growth of *M. posidonica* IVIA-Po-181^T^, which can tolerate NaCl concentrations up to 10%. It grows in the range of 5-30 ºC, is strictly aerobic and chemoorganotrophic. It does not hydrolyze starch, agar, gelatin or Tween 80. It utilizes D-glucose, D-galactose, D-fructose, sucrose, maltose, D-sorbitol, L-glutamate, malate and acetate as carbon sources.

**Table 1:** Classification and general features of *Marinomonas posidonica* IVIA-Po-181^T^ according to the MIGS recommendations [6]

**MIGS ID**	**Property**	**Term**	**Evidence code**^a^
	Current classification	Domain *Bacteria*	TAS [7]
		Phylum *Proteobacteria*	TAS [8]
		Class *Gammaproteobacteria*	TAS [9,10]
		Order *Oceanospirillales*	TAS [9,11]
		Family *Oceanospirillaceae*	TAS [9,12]
		Genus *Marinomonas*	TAS [13-15]
		Species *Marinomonas posidonica*	TAS [2]
		Type strain IVIA-Po-181^T^, CECT 7376 ^T^	TAS [2]
	Gram stain	Negative	TAS [2]
	Cell shape	Helical	TAS [2]
	Motility	Single polar flagellum	TAS [2]
	Sporulation	none	TAS [2]
	Temperature range	5 ºC-30º C	TAS [2]
	Optimum temperature	25º C	
	Carbon source	Carbohydrates, amino acids	TAS [2]
	Energy source	Chemoheterotroph	TAS [2]
	Terminal electron receptor	Oxygen	TAS [2]
MIGS-6	Habitat	Sea water	TAS [2]
MIGS-6.3	Salinity	0.5-10% NaCl	TAS [2]
MIGS-22	Oxygen	Aerobic	TAS [2]
MIGS-15	Biotic relationship	Microbiota of the rhizome of *Posidonia oceanica*	TAS [2]
MIGS-14	Pathogenicity	None	TAS [2]
MIGS-4	Geographic location	Pollença Bay, Balearic Islands in the Mediterranean Sea	TAS [2]
MIGS-5	Sample collection time	May 2005	IDA
MIGS-4.1	Latitude	N 39,89	IDA
MIGS-4.2	Longitude	E 3,09	IDA
MIGS-4.3	Depth	4 m	IDA
MIGS-4.4	Altitude	not reported	

Evidence codes - IDA: Inferred from Direct Assay; TAS: Traceable Author Statement (i.e., a direct report exists in the literature); NAS: Non-traceable Author Statement (i.e., not directly observed for the living, isolated sample, but based on a generally accepted property for the species, or anecdotal evidence). These evidence codes are from the Gene Ontology project [16]. For the purposes of this specific publication, if the evidence code is IDA, then the property was observed on a live isolate by one of the authors, or an expert mentioned in the acknowledgements.

## Genome sequencing information

### Genome project history

The genus *Marinomonas* comprises an increasing number (21 as of March 2012) of recognized bacterial species isolated from different marine habitats, including the seagrass *Posidonia oceanica* [1,2]. *Marinomonas* species show important differences in terms of substantive phenotypic characteristics. For instance, the cells of some species are helical, while others are either straight or curved rods [2]. *M. mediterranea,* which can be also isolated from *P. oceanica* [1], is the only known species within the genus that expresses a tyrosinase involved in melanin synthesis [17]. Another unique feature of that strain is the synthesis of a multicopper oxidase with laccase activity [18]. A different strain, *Marinomonas sp.* MWYL1, can catabolize dimethylsulfoniopropionate (DMSP), releasing a gas, dimethyl sulfide (DMS), which has a range of environmental effects, including changes in animal behavior and sulfur biogeochemical cycling [19,20]. *M. posidonica* is an abundant component of the culturable microbiota associated with *P. oceanica*. Since this seagrass is of enormous importance in Mediterranean ecosystems, it would be interesting to determine the metabolic capacities of the associated microorganisms. In addition, the genomic comparison with other genome-sequenced *Marinomonas* strains, namely *M. mediterranea* MMB-1^T^ [21] and *Marinomonas sp.* MWYL1 (accession number NC-009654), could offer clues about the diverse properties of this marine genus.

The *M. posidonica* IVIA-Po-181^T^ genome was sequenced under the Community Sequencing Program, CSP-2010 of DOE Joint Genome Institute who performed the sequencing, finishing and annotation. The genome has been deposited in GenBank with accession number NC_015559. Table 2 presents the project information and its association with MIGS version 2.0 compliance [6].

**Table 2:** Project information

**MIGS ID**	**Property**	**Term**
MIGS-31	Finishing quality	Finished
MIGS-28	Libraries used	Three genomic libraries: one Illumina GAii shotgun library, one 454 Titanium standard library, one paired-end 454 library
MIGS-29	Sequencing platforms	Illumina GAii, 454 GS FLX Titanium
MIGS-31.2	Fold coverage	746.4 × Illumina, 37.9 × 454
MIGS-30	Assemblers	Newbler version 2.3, Velvet version .7.63, phrap version SPS 4.24
MIGS-32	Gene calling method	Prodigal, GenePRIMP
	Genome Database release	February 19, 2011
	Genbank ID	NC_015559
	Genbank Date of Release	May 24, 2011
	GOLD ID	Gc01770
	NCBI project ID	52545
MIGS-13	Source material identifier	CECT 7376^T^, NCIMB 14433^T^
	Project relevance	Comparative analysis, Environmental

### Growth conditions and DNA isolation

In order to isolate quality genomic DNA for sequencing, *Marinomonas posidonica* IVIA-Po-181^T^ was grown from a -70 ºC stock in MMC agar medium [22]. A single colony was inoculated into the same broth medium and incubated at 25 ºC overnight. This culture was used to inoculate 200 ml of MMC at OD_600_ 0.05. The culture was grown at 25 ºC, shaking at 130 rpm to early stationary phase (OD_600_ 0.75), when it was kept at 4 ºC for 20 minutes to halt replication. DNA isolation from this culture was performed using the CTAB method (Ausubel *et al*., 1994) with some modifications. The cells were harvested by centrifugation (6000 × g) and the pellet resuspended in T_10_E_1_ pH 8 to an OD_600_ of 1.0. The cell suspension was treated at 37 ºC for 30 minutes with 0.53% SDS (Sigma) and 0.1 mg/ml Proteinase K (Fermentas). After the addition of RNase A (DNase-free from Qiagen) at 0.01 mg/ml, the cells were incubated at 37 ºC for another 30 min. To remove cell wall debris, denatured proteins and polysaccharides, the NaCl concentration was raised to 0.6 M, and 28.5 mM of CTAB (preheated to 65 ºC) was added to the cell extract, followed by incubation for 10 minutes at 65 ºC. CTAB-protein and CTAB-polysaccharide complexes were removed by chloroform/isoamyl alcohol (24:1), followed by phenol/chloroform/isoamyl alcohol (25:24:1) extractions. To precipitate the nucleic acids in the aqueous phase, 0.6 vol of isopropanol was added and the sample was incubated for 30 minutes at room temperature. The DNA precipitate was recovered by centrifugation, followed by a 70% ethanol wash to remove residual CTAB. The DNA pellet was resuspended in TE with 0.1 mg/ml RNaseA and stored at -80 ºC until further used.

### Genome sequencing and assembly

The draft genome of *Marinomonas posidonica* IVIA-Po-181^T^ was generated at the DOE Joint Genome Institute (JGI) using a combination of Illumina [23] and 454 technologies [24]. For this genome, we constructed and sequenced an Illumina GAii shotgun library which generated 58,265,190 reads totaling 4,428.2 Mb, a 454 Titanium standard library which generated 252,750 reads and one paired end 454 library with an average insert size of 7 kb which generated 709,174 reads totaling 203.5 Mb of 454 data. All general aspects of library construction and sequencing performed at the JGI can be found at the JGI website [25]. The initial draft assembly contained 35 contigs in 1 scaffold. The 454 Titanium standard data and the 454 paired end data were assembled together with Newbler, version 2.3. The Newbler consensus sequences were computationally shredded into 2 kb overlapping fake reads (shreds). Illumina sequencing data were assembled with VELVET, version 0.7.63 [26], and the consensus sequences were computationally shredded into 1.5 kb overlapping fake reads (shreds). We integrated the 454 Newbler consensus shreds, the Illumina VELVET consensus shreds and the read pairs in the 454 paired end library using parallel phrap, version SPS - 4.24 (High Performance Software, LLC). The software Consed [27-29] was used in the following finishing process. Illumina data were used to correct potential base errors and increase consensus quality using the software Polisher developed at JGI (Alla Lapidus, unpublished). Possible mis-assemblies were corrected using gapResolution (Cliff Han, unpublished), Dupfinisher [30], or by sequencing cloned bridging PCR fragments with sub-cloning. Gaps between contigs were closed by editing in Consed, by PCR and by Bubble PCR (J-F Cheng, unpublished) primer walks. A total of 110 additional reactions were necessary to close gaps and to raise the quality of the finished sequence. The total size of the genome is 3,899,940 bp and the final assembly is based on 130.3 Mb of 454 draft data, which provide an average 33.4× coverage of the genome and 4,327.5 Mb of Illumina draft data, which provide an average 1,109.6× coverage of the genome.

### Genome annotation

Genes were identified using Prodigal [31] as part of the Oak Ridge National Laboratory genome annotation pipeline, followed by a round of manual curation using the JGI GenePRIMP pipeline [32]. The predicted CDSs were translated and used to search the National Center for Biotechnology Information (NCBI) non-redundant database, UniProt, TIGRFam, Pfam, PRIAM, KEGG, COG, and InterPro databases. These data sources were combined to assert a product description for each predicted protein. Non-coding genes and miscellaneous features were predicted using tRNAscan-SE [33], RNAMMer [34], Rfam [35], TMHMM [36], and signalP [37]. Further comparative analysis was performed using the IMG-ER system [38].

## Genome properties

**Figure 3:**
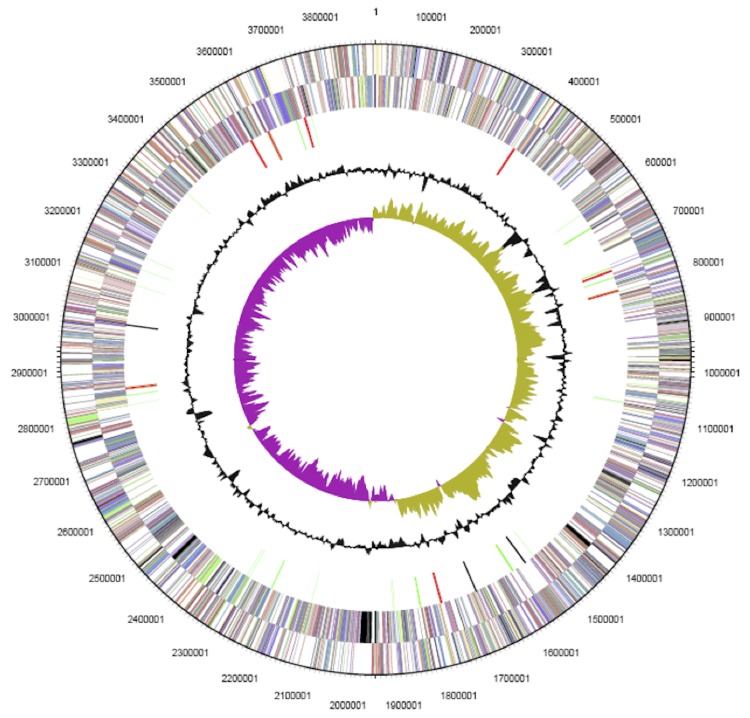
Graphical circular map of the chromosome. From outside to the center: genes on forward strand (color by COG categories), genes on reverse strand (color by COG categories) RNA genes (tRNAs green, rRNAs red, other RNAs black), GC content, GC skew.

The genome has no plasmids, and the 3,899,940 bp circular chromosome has a GC content of 44.29% (Table 3 and Figure 3). Of the 3,651 predicted genes, 3,544 were protein-coding genes, and 107 RNAs. There were 53 pseudogenes. The majority of the protein-coding genes (78.09%) were assigned with a putative function; the others were annotated as hypothetical proteins. The distribution of genes into COGs functional categories is presented in Table 4.

**Table 3:** Genome statistics

**Attribute**	**Value**	**% of total^a^**
Genome size (bp)	3,899,940	100
DNA coding region (bp)	3,492,581	89.55
DNA G+C content (bp)	1,727,094	44.29
Number of replicons	1	
Extrachromosomal elements	0	
Total genes	3,651	100.00
Pseudogenes^b^	53	1.45
RNA genes	107	2.93
rRNA operons	8	
Protein-coding genes	3,544	97.07
Genes with function prediction	2,851	78.09
Genes in paralog clusters	1,734	47.49
Genes assigned to COGs	3,062	83.87
Genes with Pfam	3,096	84.80
Genes with signal peptides	702	19.23
Genes with transmembrane helices	852	23.34
CRISPR repeats	0	

a) The total is based on either the size of the genome in base pairs or the total number of protein coding genes in the annotated genome.

b) Pseudogenes may also be counted as protein-coding or RNA genes, so are not additive under total gene count.

**Table 4:** Number of genes associated with the 25 general COG functional categories

**Code**	**Value**	**% age**	**Description**
J	181	5.26	Translation, ribosomal structure and biogenesis
A	1	0.03	RNA processing and modification
K	289	8.4	Transcription
L	119	3.46	Replication, recombination and repair
B	2	0.06	Chromatin structure and dynamics
D	31	0.9	Cell cycle control, cell division and chromosome partitioning
Y	0	0	Nuclear structure
V	22	0.64	Defense mechanisms
T	254	7.38	Signal transduction mechanisms
M	157	4.56	Cell wall/membrane/envelope biogenesis
N	97	2.82	Cell motility
Z	0	0.0	Cytoskeleton
W	0	0.0	Extracellular structures
U	75	2.18	Intracellular trafficking, secretion and vesicular transport
O	129	3.75	Post-translational modification, protein turnover, chaperones
C	175	5.09	Energy production and conversion
G	239	6.95	Carbohydrate transport and metabolism
E	366	10.64	Amino acid transport and metabolism
F	77	2.24	Nucleotide transport and metabolism
H	165	4.8	Coenzyme transport and metabolism
I	109	3.17	Lipid transport and metabolism
P	195	5.67	Inorganic ion transport and metabolism
Q	86	2.5	Secondary metabolites biosynthesis, transport and catabolism
R	372	10.81	General function prediction only
S	299	8.69	Function unknown
-	589	16.13	Not in COGs

## Insights from genome sequence

**Figure 4:**
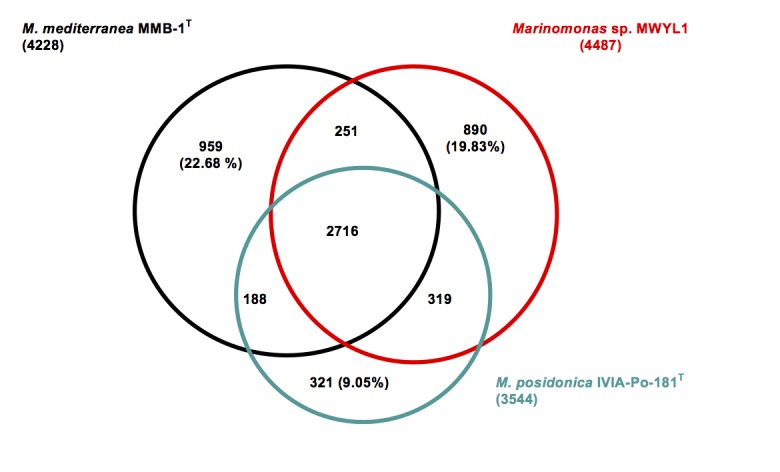
Venn-diagram showing the intersection of proteins sets (total number in parenthesis) of the three completed *Marinomonas* genomes. The intersections were calculated using the IMG tool phylogenetic profiler with default values (E value 1 e^-5^ and 30% minimum identity). All intersections concerning *M. posidonica* IVIA-Po-181^T^ are gene counts in this strain. The remaining intersection between *M. mediterranea* and *Marinomonas sp.* MWYL1 represents gene counts in *M. mediterranea*. In some cases, the intersections do not add to total numbers of genes, due to the dependence of the results on the strain used as query.

The genome of *M. posidonica* IVIA-Po-181^T^ (3.9 Mb) is 16.75 and 23.54% smaller than those of *M. mediterranea* (4.6 Mb) and *Marinomonas sp.* MWYL1 (5.1 Mb) respectively. Comparison of the protein sets of these three *Marinomonas* strains revealed that the genome of IVIA-Po-181^T^ encodes a much smaller number of unique proteins (Figure 4), indicating that most of the proteins encoded in its genome are also present in the other two strains. Among these is a cluster involved in the catabolism of DMSP resulting in the production of DMS, an environmentally important gas. This capacity was first described in *Marinomonas sp.* MWYL1, where the genes involved are Mmwyl1_4041- Mmwyl1_4045 [20]. Highly similar clusters of genes are detected in *M. mediterranea* MMB-1^T^ (Marme_2354-Marme_2350) and *M. posidonica* IVIA-Po-181^T^ (Mar181_0285-Mar181_0290). Although DMSP is an intracellular compatible solute synthesized by marine phytoplankton, as far as we know, its synthesis by *P. oceanica* has not been reported.

The analysis of the set of unique genes in *M. posidonica* IVIA-Po-181^T^ revealed that 57% (183 out of 321) of such genes are annotated as encoding hypothetical proteins. From Mar181_0471 to Mar181_0507 there is a cluster of genes whose sequence suggests that they are involved in the synthesis of exopolysaccharide. It is tempting to speculate that such a polymer might be involved in cellular attachment to the plant. Two other genes of interest are the adjacent Mar181_1314, annotated as coding for a insecticidal toxin complex/plasmid virulence protein, and Mar181_1315 annotated as encoding RHS repeat-associated core domain, both of these gene products being very large polypeptides, of 2,746 and 2,149 amino acids respectively. BLAST-based searches using each of these as the query, showed marked similarities to proteins that are in the insecticidal protein complex (Tc) synthesized by different entomopathogenic bacteria, including endosymbionts of nematodes [39].

The product of Mar181_1315 seems to be the result of a fusion of different proteins. The first 1,500 amino acids show 29.4% identity and 45.3% similarity to *Photorhabdus luminescens* TcdB1 (accession number AAL18467) [40]. Both proteins also show some similarity to the pathogenic factor SpvB of *Salmonella enterica.* However, the similarity is limited to the N terminal region and does not include the region from amino acids 375-391 of SpvB which encodes its ADP-ribosyl transferase activity [41].

In addition, the product of Mar181_1315 shows, from amino acid 1,400 to 2,141, similarity to both, the toxin protein TccC2 and Rhs elements, consistent with the similarity of Rhs proteins to some toxin proteins, such as TccC2, which was also previously noticed [40]. For instance, the product of Mar181_1315 shows the typical conserved sequences limiting the core of Rhs proteins [42]. The lower limit is R(1412)VxxxxxxxG and the upper limit is P(2033)XXXXDPXGL. *rhs-*like genes are distributed among many different bacteria, the genetic acronym “*rhs*” being originally based on “recombination hot spots” since they were initially related to genomic rearrangements [43]. However, they are now considered to be involved in many different cellular processes. For example, it has been recently shown that they show high similarity to the pairs of proteins CdiA/CdiB involved in contact-dependent growth inhibition and toxin/immunity function [44]. In relation to the similarity to TccC2 (accession number AAL18492), part of the Mar181_1315 product (from amino acid N(1467) to G(2141)) displays 34% identity and 49% similarity to the first 663 amino acids of *P. lumincescens* TccC2. Finally, the C-terminal 250 amino acids of the Mar181_1315 do not show similarity to any protein in the NCBI database. The variability of the C-terminal region is also a property of Rhs proteins [42].

It is noteworthy that many different proteins showing an organization similar to the product of Mar181_1315 occur in a wide range of taxonomically distinct bacteria such as *Wolbachia* , an endosymbiont of *Cadra cautella* (BAH22314), the myxobacterium *Sorangium cellulosum* (YP001619210) or the Gram-positive *Paenibacillus curdlanolyticus* (ZP_07386246). There are even homologues in some ascomycete fungi such as the plant pathogenic fungus *Verticillum dahliae* (EGY15726) and *Podospora anserina* (XP_001907253).

The locus Mar181_1314, encoding a protein of 2,746 amino acids is also similar to proteins that form part of insecticidal complexes such as TcdA1 (AAL18486) from *Photorhabdus luminescens* [40]. Genes encoding proteins similar to Mar181_1314 are detected in diverse microorganisms, and interestingly, in many cases they are close to the genes encoding proteins similar to Mar181_1315, for instance in *Wolbachia* (BAH22316), *S. cellulosum* (YP001619211), *P. curdlanolyticus* (ZP_07386247) and even the fungus *P. anserina* (XP_001907253), as well as in *Photorhabdus luminescens.* The expression of both proteins in *M. posidonica* would determine that all the components necessary for the toxicity of the toxin complexes are present [40]. It is remarkable that there are no detectable homologues of either gene product in any other *Marinomonas* or indeed, in any genome-sequenced strain of the *Oceanospirillales*.

The small number of unique genes in *M. posidonica* IVIA-Po-181^T^ could be related to the observation that this strain has fewer mobile genetic elements than the other genome-sequenced *Marinomonas* strains, as deduced by the numbers of genes encoding predicted transposases or inactive derivatives; there are 44 in *M. mediterranea* MMB-1^T^, 33 in *Marinomonas sp.* MWYL1, but only 6 in *M. posidonica* IVIA-Po-181^T^. Furthermore, the genome of strain IVIA-Po-181^T^ does not apparently contain any prophages, but there are at least two in both strain MMB-1^T^ and strain MWYL1. In relation to CRISPR repeats, which are involved in phage resistance, there are two complete CRISPR sequences in *Marinomonas sp.* MWYL1 and four such elements in *M. mediterranea*, two of which are complete and two that lack Cas associated proteins. On the contrary, no CRISPR elements are detected in *M. posidonica*.

The genome size of *M. posidonica* IVIA-Po-181^T^ is in the lower range of those observed in marine bacteria of the copiotrophs/opportunitroph type, which are characterized by a high growth rate when nutrients become available [45,46]. The smaller size of the genome of *M. posidonica* compared to the other *Marinomonas* strains could be a consequence of a close and stable relationship with *Posidonia oceanica*, making unnecessary a high genomic potential for the adaptation to different environments.

The availability of the genome sequence of this strain may facilitate direct examination of the functions that are involved in its association with one of the more important marine plant species.
